# Creep and cracking of concrete hinges: insight from centric and eccentric compression experiments

**DOI:** 10.1617/s11527-017-1112-9

**Published:** 2017-11-22

**Authors:** Thomas Schlappal, Michael Schweigler, Susanne Gmainer, Martin Peyerl, Bernhard Pichler

**Affiliations:** 1 Institute for Mechanics of Materials and Structures, TU Wien – Vienna University of Technology, Karlsplatz 13/202, 1040 Vienna, Austria; 2 Smart Minerals GmbH, Reisnerstraße 53, 1030 Vienna, Austria

**Keywords:** Integral bridge construction, Mechanized tunneling, Segmented tunnel lining, Tensile cracking of concrete, Digital image correlation

## Abstract

Existing design guidelines for concrete hinges consider bending-induced tensile cracking, but the structural behavior is oversimplified to be time-independent. This is the motivation to study creep and bending-induced tensile cracking of initially monolithic concrete hinges systematically. Material tests on plain concrete specimens and structural tests on marginally reinforced concrete hinges are performed. The experiments characterize material and structural creep under centric compression as well as bending-induced tensile cracking and the interaction between creep and cracking of concrete hinges. As for the latter two aims, three nominally identical concrete hinges are subjected to short-term and to longer-term eccentric compression tests. Obtained material and structural creep functions referring to *centric* compression are found to be very similar. The structural creep activity under *eccentric* compression is significantly larger because of the interaction between creep and cracking, i.e. bending-induced cracks progressively open and propagate under sustained eccentric loading. As for concrete hinges in frame-like integral bridge construction, it is concluded (i) that realistic simulation of variable loads requires consideration of the here-studied time-dependent behavior and (ii) that permanent compressive normal forces shall be limited by 45% of the ultimate load carrying capacity, in order to avoid damage of concrete hinges under sustained loading.

## Introduction

Concrete hinges were invented by Freyssinet [1, 2]. They are unreinforced or marginally reinforced necks in reinforced concrete structures, such as, e.g. supports in integral bridge construction [3–10] and segment-to-segment interfaces of segmented linings used in mechanized tunneling [11–28]. Eventually a few pairs of crossed steel rebars (or bolts) run across a concrete hinge. Their cross-over point is typically at the center of the neck. Therefore, the bending stiffness of the neck is significantly smaller than the ones of the two connected reinforced concrete parts. The corresponding concentration of bending deformation at the concrete hinge results, already under regular service loads, either in tension-induced cracking of initially monolithic necks, or in partial separation of segment-to-segment interfaces. Both effects further reduce the effective bending stiffness of the neck. This further promotes the ability of concrete hinges to develop relative rotation angles.

**Fig. 1:**
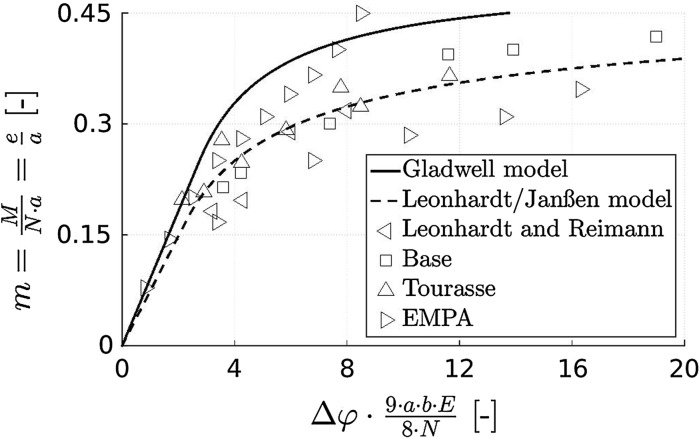
Dimensionless relation between bending moment *M* and relative rotation angle $\Delta \varphi$ (“Leonhardt and Reimann”-diagram): modeled relationships by Gladwell [30], Leonhardt and Reimann [5], and Janßen [17], as well as experimental data from Base [33], Tourasse [34], and EMPA [35]; $N=$ normal force, $e=$ eccentricity, $a=$ width of neck, $b=$ depth of neck, $E=$ Young’s modulus of concrete

Current design guidelines for concrete hinges are based on pioneering developments of Leonhardt and Reimann [5]. In the field of integral bridge construction, these guidelines were further developed by Mönnig and Netzel [29], Marx and Schacht [6], as well as by Morgenthal and Olney [7]. As for mechanized tunneling, Gladwell [30] developed a moment-rotation relation for concrete hinges representing longitudinal joints. A few years later, Janßen [17] adapted the formulas of Leonhardt and Reimann to interfaces between reinforced concrete segments. These formulas are up to date the golden standard [13, 27, 28, 31, 32]. All described guidelines and recommendations provide unique relationships between bending moment and rotation angle, see their comparison with available experimental data in Fig. 1.

Concrete hinges are nowadays experiencing a renaissance in integral bridge construction. Examples are (i) the viaduct “Weißenbrunn am Forst”, Germany, which was constructed in 2011, see [36], and (ii) the Huyck-bridge, Austria, which was finished in 2014, see [37].

Since the 1960s, many concrete hinges were investigated experimentally, e.g., by Tourasse [34], Base [33], Dix [38], Leonhardt and Reimann [5], Fessler [35], Franz and Fein [39], as well as Hordijk and Gijsbers [40]. The underlying test protocols are typically structured in two phases. At first, a compressive axial force was applied and kept constant thereafter. This was followed by imposing a rotation angle and keeping it constant thereafter. The first type of loading resulted in *creep* of concrete, and the second type in *stress relaxation*. This mixed viscoelastic behavior of concrete was one motivation for the current contribution, where we focus on *creep* of concrete and of concrete hinges subjected to compression and bending. The second motivation is that the interaction between bending-induced tensile cracking and creep of initially monolithic concrete hinges is an open research question.

We report on experiments both on the material level of plain concrete and on the structural scale of initially monolithic concrete hinges. As for material testing, concrete prisms are subjected to centric compression, in order to quantify material creep functions at load levels being equal to virtually 20% of the short-term uniaxial compressive strength. As for structural testing, initially monolithic concrete hinges are subjected to centric and eccentric compression. The underlying aim is to study (i) structural creep under centric loading, (ii) tensile cracking under short-term eccentric loading, (iii) structural creep under eccentric compression at virtually 25% of the ultimate load carrying capacity, and (iv) the interaction between bending-induced tensile cracking and creep. Rotations of the concrete hinges are quantified by means of Inductive Displacement Transducers (LVDTs). Crack propagation is observed with a non-contact displacement measurement system, based on Digital Image Correlation (DIC).

The present contribution is structured as follows. Materials and test specimens are discussed in Sect. 2. Characterization of linear creep of plain concrete under uniaxial compression is the topic of Sect. 3. Structural testing of concrete hinges up to service loads as well as up to their load carrying capacity are described in Sects. 4 and 5, respectively. Experimental results are analyzed in Sect. 6 and discussed in Sect. 7, including implications for design guidelines and for the necessary consideration of time-dependent behavior of concrete hinges in integral bridge construction. Conclusions are drawn in Sect. 8. Throughout this work, compression is considered by a positive sign.

## Materials and test specimens

### Concrete and steel

Concrete C 35/45 F45 GK16 B5 [41] is produced with a commercial CEM II/A-L 42.5 N cement [41], Viennese tap water, and calcite aggregates with a maximum size of $a_\mathrm{agg}=16$ mm. The initial water-to-cement mass ratio amounts to $w/c=0.48$, the initial aggregate-to-cement mass ratio to $a/c=3.97$, and the initial mass density to $\rho =2318$ kg/m$^3$. The cube compressive strength $f_{c,\mathrm{cube}}$ and Young’s modulus *E* are determined 28 days after production, following the Austrian standards for testing of concrete [42], as

1 $$\begin{aligned} f_{c,{\mathrm{cube}}}=56.25\,{\text {MPa}},\quad E=34.75\,{\text {GPa}}. \end{aligned}$$

The cube compressive strength allows for estimating the uniaxial compressive strength $f_c$ as [43]

2 $$\begin{aligned} f_c = \frac{f_{c,{\mathrm{cube}}}}{1.2} = 46.88\,{\text {MPa}}. \end{aligned}$$

The produced concrete is well suited for bridge applications. In order to provide adequate frost-thaw resistance within exposure class XF3, it contains 2.5–5.0 volume percent entrained air.

As for the rebars, steel quality B550 A was chosen. The von Mises yield stress $f_y$ and Young’s modulus $E_s$ of this material amount to

3 $$\begin{aligned} f_y=550\,{\text{MPa}},\quad E_s=200\,{\text {GPa}}. \end{aligned}$$

### Plain concrete prisms and reinforced concrete hinges

**Fig. 2:**
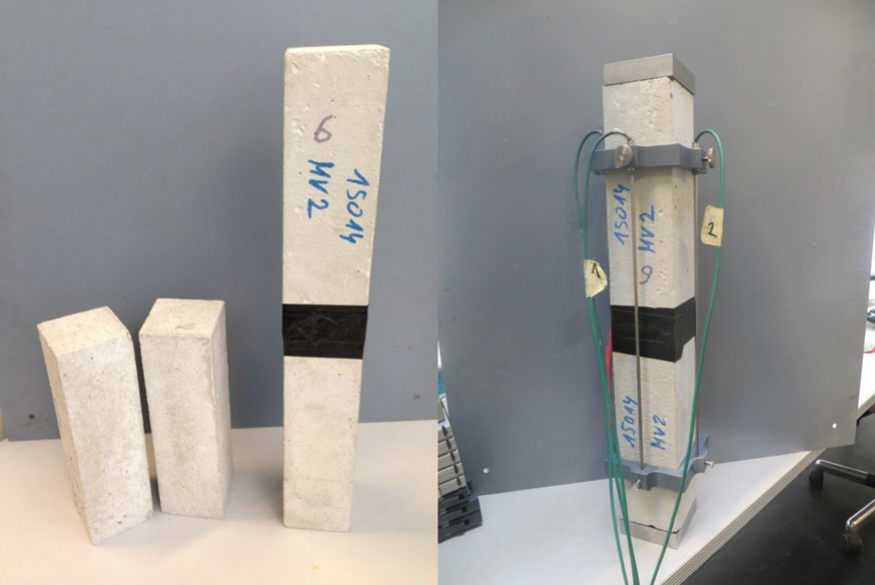
Plain concrete prisms used for material testing: test specimens consist of two prisms in serial arrangement

As for material testing of plain concrete, 10 prisms are produced. They exhibit nominal dimensions of 75 mm $\times$ 75 mm $\times$ 250 mm, see Fig. 2.

**Fig. 3:**
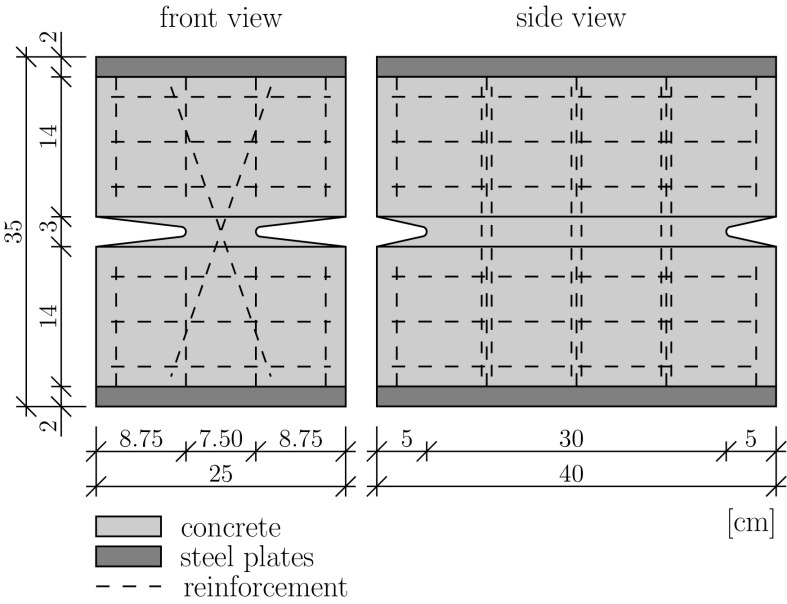
Geometric dimensions and rebar positions of the tested reinforced concrete hinges

As for structural testing, three marginally reinforced concrete hinges are produced. Their geometric shape (i) is inspired by the concrete hinges of Huyck-bridge [37], and (ii) complies with the design guidelines of Leonhardt and Reimann [5]. In more detail, the concrete hinges exhibit width, height, and depth amounting to 25, 35, and 40 cm, respectively, see Fig. 3. Lateral notches are 8.75 cm deep and front notches 5 cm, such that the cross-sectional area of the neck, $A_c$, amounts to

4 $$\begin{aligned} A_c = 7.5\times 30 = 225.00\,\text {cm}^2. \end{aligned}$$

Both at the top and at the bottom of the concrete hinges, a steel plate of 2 cm thickness is welded (well before casting of fresh concrete) to the neighboring reinforcement cage (Fig. 3). These plates ensure an effective distribution of concentrated external line loads. The top and bottom reinforcement cages are connected by three pairs of crossed steel rebars, with cross-over points right at the center of the neck, see Fig. 3. They exhibit a diameter of 0.8 cm, such that the total cross-sectional area of reinforcements running across the neck, $A_s$, amounts to

5 $$\begin{aligned} A_s=6 \times 0.8^2 \pi /4=3.02\,\text {cm}^2. \end{aligned}$$

The reinforcement ratio $\varrho$ follows as

6 $$\begin{aligned} \varrho =A_s/A_c=1.3\%. \end{aligned}$$

Three batches of fresh concrete are mixed in a laboratory mixer. From each batch, two to four plain concrete prisms and one reinforced concrete hinge are produced at the same time. Formworks are stripped 24 h after production. After that, all specimens are air cured in order to simulate a practical real-life application. In other words, the specimens were allowed to dry in an environment with temperature und relative humidity ranging in estimated intervals from 24 to 29$\,^\circ$C and from 50 to 70 %, respectively.

## Material testing: creep characterization of plain concrete

Plain concrete exhibits *linear creep* as long as the stress level $\sigma$ is small compared to the uniaxial compressive strength $f_c$, and *nonlinear creep* for larger stress levels. The transition from linear to nonlinear creep is still not fully understood. Eurocode 2—regulating the design of concrete bridges, see [44]—suggests that the transition threshold amounts to $\sigma /f_c = 0.45$ while Mazzotti and Savoia [45] recommend a more conservative value amounting to $\sigma /f_c = 0.20$.

This is the motivation to perform five linear creep tests with compressive stress level $\sigma \approx 0.2\,f_c$, on an electromechanical testing machine of type Zwick/Roell Z050. Loading is applied via two metal prisms with a cross-section of 75 $\times$ 75 mm$^2$, and a height of 20 mm, see Fig. 2.

Compression-induced shortening of the specimens is measured directly at the surface of the specimens. In order to increase the measurement length, two concrete prisms are put into a serial arrangement, see Fig. 2 and Table 1. To this end, the two prisms are pushed together by hand and the prism-to-prism interface is connected laterally using an adhesive tape (Fig. 2). The shortening of the specimens is measured by four Inductive Displacement Transducers (LVDTs) of type HBM W1/2mm-T. They are mounted to the specimen by means of LVDT holders which are attached to the specimen by means of screws. The LVDT holders are located at a distance of 75 mm to the top and bottom load platens (Fig. 2), i.e. they measure the shortening of the specimen in the central region which is free of friction-induced, unavoidable, and self-equilibrated shear stresses activated in the interfaces between specimen and load platens, see [46] for more details. Firm contact in the prism-to-prism interface avoided possible sliding or rotation/bending of the prisms. This is underlined by the four individual LVDT measurements which show no indication of a significant eccentricity of loading.

**Table 1:** Properties of plain concrete specimens used for characterization of linear creep behavior

No.	Height *h* (mm)	Cross-section $a_1\times a_2$ (mm$^2$)	Mass *m* (kg)	Mass density $\rho$ (kg/m$^3$)
1	501.10	75.48 × 76.83	6.794	2338.27
2	500.90	75.55 × 75.48	6.706	2347.87
3	501.50	75.60 × 76.05	6.752	2341.75
4	501.50	75.43 × 76.33	6.764	2342.88
5	500.65	74.98 × 75.40	6.672	2357.40

In order to avoid temperature-induced deformation of the specimens during creep testing, the experiments are carried out in an insulated test chamber conditioned to 20 °C with a temperature control unit Lauda RK8 KP. In addition, after closing the test chamber and before starting the creep tests, specimens are allowed to achieve isothermal conditions during a waiting period amounting to more than 24 h.

The creep tests are carried out as follows. All of the five specimens are involved in a linear creep test. During the described waiting period before actual loading, the specimens are subjected to a compressive force of 2 kN, in order to establish good contact along the interfaces between load platens and specimen as well as the prism-to-prism interface in the middle of the specimen. Compressive loading is increased with a force rate amounting to 1 kN/s up to 49 kN, and the load level $\sigma \approx 0.2\,f_c$ is kept constant for 20 h (specimens 1, 2, and 3) and for 12 h (specimens 4 and 5), respectively.

## Structural testing of concrete hinges up to service loads

A first set of structural tests deals with creep and tensile cracking of concrete hinges subjected to centric and eccentric service loads. The test setup is described in Sect. 4.1. The extensional stiffness of undamaged concrete hinges and their structural creep behavior under centric compression is the topic of Sect. 4.2. The bending stiffness of undamaged concrete hinges and the development of tensile cracking as a function of eccentric loading is studied in Sect. 4.3. Finally, the coupling between creep and tensile cracking of concrete hinges is analyzed in eccentric creep tests, see Sect. 4.4.

### Test setup and measurement equipment

Concrete hinges are subjected, 26 days after production, to compressive line loads using a servo-hydraulic testing machine of the type Walter and Bai DLFV-250DZ-10-D. The load measurement cell of the testing machine is used to carry out force-controlled tests, where compressive normal forces *N* are imposed on the concrete hinges. Tests are carried out with and without eccentricity of the normal force. In case of load eccentricity *e*, concrete hinges are subjected to coupled compression *and* bending, whereby the bending moment *M* is proportional to the normal force:

7 $$\begin{aligned} M = N\cdot e. \end{aligned}$$

**Fig. 4:**
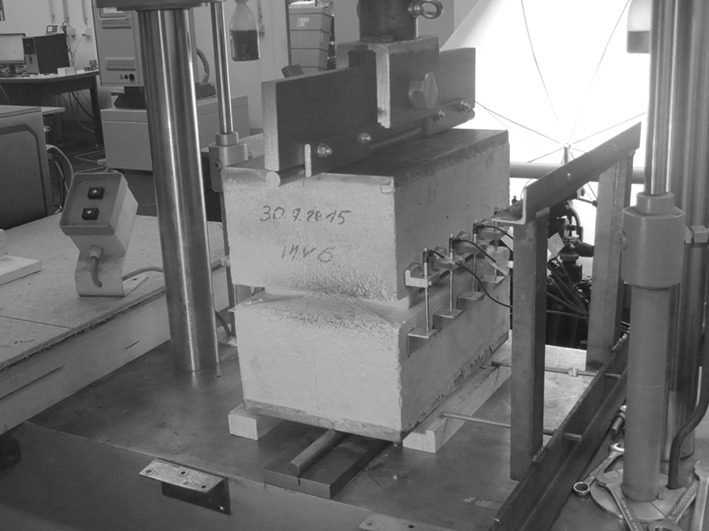
Structural testing of concrete hinges up to service loads: Five Inductive Displacement Transducers are mounted on each lateral surface of the concrete hinges; two pairs of cameras of a Digital Image Correlation system are used to monitor tensile cracking at the notch roots of the front side and the back side notches (not shown); normal forces *N* are imposed in form of line loads, acting with eccentricity *e*, resulting in combined compression and bending, see also Eq. (7); temporary supports are removed during testing

Ten LVDTs of type HBM W1/2mm-T are mounted to the lateral surfaces of the concrete hinges, in order to measure changes of the notch mouth opening displacements of the lateral notches, see Fig. 4. The measurement length of the displacement sensors, measured in loading direction, amounts to $\ell _\varepsilon =65\,\mathrm {mm}$. The LVDTs are arranged symmetrically in thickness direction with a sensor-to-sensor distance amounting to 80 mm. The measurement axis of each LVDT exhibits a normal distance of 22 mm to the lateral surface of the concrete hinge, i.e. opposite LVDTs exhibit a mutual distance which amounts to $\ell _{\varphi } =250\,\mathrm {mm}+2\cdot 22\,\mathrm {mm}=294\,\mathrm {mm}$.

A Digital Image Correlation (DIC) system of type Dantec Dynamics Q 400, is used to observe tensile crack propagation along the notch roots of the front side and the back side notches. To this end, both notches are monitored with a pair of 5 megapixel cameras each. Application of two cameras per notch allows for recording three-dimensional displacement fields, whereby the in-plane displacements are of special interest. The observed field of view is approximately equal to 12 cm $\times$ 10 cm. Fine speckle patterns are sprayed to the notch roots prior to testing, with a desired point size amounting to 2 to 3 pixels. A facet size of 21 pixel and grid spacing of 17 pixel are chosen, for further specifications of the DIC see Table 2.

**Table 2:** Specifications and settings used for the DIC measurements and evaluation, respectively

Dantec Dynamics Q-400 ISTRA 4.4.2	
Specifiations	
Number of 5 megapixel cameras used	$2 \times 2$
Names of front side cameras	Cam1/2
Names of back side cameras	Cam3/4
Cam1-to-Cam2 distance	60 cm
Cam3-to-Cam4 distance	60 cm
Camera-to-specimen distance	82 cm
Exposure Cam1/2	Red light
Exposure Cam3/4	Diffuse light
Facet size	21 pixel
Grid spacing	17 pixel
Object lens	2.8/50
F-number	5.6–11
Shutter speed	80 ms
Settings for evaluation	
Crack identification: upper threshold	0.006 [–]
Crack identification: lower threshold	0.002 [–]
Contour smoothing:	Disabled
Displacement smoothing:	Spline
Grid reduction factor	1
Smoothness factor	−0.5

### Creep under centric compression (200 kN)

Centric compression tests up to 200 kN are used to characterize the extensional stiffness of undamaged concrete hinges and their structural creep behavior under pure normal force (no bending). To this end, each of the three concrete hinges is installed into the testing machine, resting on temporary supports. After fine tuning the position and installing the deformation measurement equipment, loading is increased up to 4 kN. This allows for removing the temporary supports, because 4 kN loading is sufficient to keep the concrete hinge in the desired position (no tipping over). Reference readings of the deformation measurement system are taken. Finally, the loading is increased with a force rate amounting to 5 kN/s up to 200 kN. This loading is kept constant for 4 h, followed by complete unloading, using again the force rate of 5 kN/s.

### Bending and tensile cracking under short-term eccentric compression up to 200 kN

Minutes-long eccentric compression tests are used to characterize (i) the bending stiffness of undamaged concrete hinges and (ii) the development of tensile cracking as a function of the eccentricity *e* and of the compressive normal force *N*. This requires a careful development of the test design described next.

As for characterization of the *undamaged* bending stiffness of the investigated concrete hinges, a trade off must be found between (i) a *large* eccentricity, resulting in relatively large bending, see Eq. (7) and, hence, in relatively large rotation angles, as well as (ii) a *small* eccentricity, ensuring that bending-induced tensile stresses in the neck region remain smaller than the tensile strength of concrete. The latter cannot be directly measured in the present case, because the test design must be finished well before the specimens reach the testing age. Therefore, the tensile strength is estimated, based on the known compressive strength $f_c$ according to Eq. (2), using the following standard relation [43]

8 $$\begin{aligned} f_t = -0.3\,{\text {MPa}}\cdot \left( \frac{f_c}{1\,{\mathrm {MPa}}}\right) ^{2/3} = -3.9\,{\text {MPa}}. \end{aligned}$$

Two-dimensional plane strain Finite Element analyses of the investigated concrete hinges provide quantitative insight into the stress concentrations resulting from loading by a normal force *N* and by a bending moment *M*, respectively. Denoting the cross-sectional area of the neck as $a\,b$ and its beam theory-related elastic section modulus as $a^2\,b/6$, the largest tensile stress in the neck region follows as

9 $$\begin{aligned} \max \sigma _{t} = 2.77\,\frac{N}{a\,b} - 2.00\,\frac{6\,M}{a^2\,b}, \end{aligned}$$

where 2.77 and 2.00 are numerically determined stress increase factors relative to the stress levels foreseen by beam theory. Specializing Eq. (9) for *M* according to Eq. (7), and the resulting expression for $a=75\,\mathrm {mm}$, for $b = 300\,\mathrm {mm}$, for the targeted maximum loading $N=200\,\mathrm {kN}$, and for an eccentricity $e=20\,\mathrm {mm}$, delivers a maximum tensile stress which is only slightly smaller than the tensile strength:

10 $$\begin{aligned} \max \sigma _{t} = -3.8\dot{2}\,{\text {MPa}}. \end{aligned}$$

In other words, Finite Element analyses suggest that a normal force $N=200\,\mathrm {kN}$, acting with an eccentricity of $e=20\,\mathrm {mm}$ on the herein investigate concrete hinges, can be expected to result in large tensile stresses, but small enough to keep the concrete hinges intact (no tensile cracking).

As for studying the development of tensile cracking as a function of the eccentricity *e* and of the compressive normal force *N*, a maximum reasonable eccentricity needs to be defined. In this context, we consider the design guidelines of Leonhardt and Reimann [5], which exhibit a limit of application reading as

11 $$\begin{aligned} \max e = a/3, \end{aligned}$$

because the guidelines envision (i) that for $e=a/3$ tensile cracking will extend across half of the neck width, i.e. right to the center of the concrete hinge, and (ii) that an even larger extension of tensile cracking shall be avoided. Specializing Eq. (11) for the neck width $a=75\,\mathrm {mm}$ of the investigated concrete hinges delivers the maximum eccentricity to amount to $e = 25\,\mathrm {mm}$. This eccentricity is chosen for the load carrying capacity tests described in Sect. 5.

As for the tests with service loads $N\le 200\,\mathrm {kN}$, three different eccentricities are chosen:

12 $$\begin{aligned} e \in \big \{20\,\mathrm {mm}\,, 22\,\mathrm {mm}\,, 24\,\mathrm {mm}\big \}. \end{aligned}$$

Testing is started with the smallest eccentricity $e=20\,\mathrm {mm}$. One after the other, the concrete hinges are installed into the testing machine, resting on temporary supports. After fine tuning the position and installing the deformation measurement equipment, loading is increased up to 0.2 kN. This allows for removing the temporary supports, because 0.2 kN loading is sufficient to keep the concrete hinge in the desired position (no tipping over). Reference readings of the deformation measurement system are taken. Right before starting the test, the DIC system is activated to take pictures with a frequency amounting to 2 Hz. Loading is increased with a force rate amounting to 5 kN/s. Once 25 kN are reached, the loading process is stopped for a waiting period of 5 s, such that the DIC system takes 10 pictures at that load level. After that, loading is increased by another 25 kN, followed by the next waiting period. Step-by-step loading is increased up to a maximum load of 200 kN. After another waiting period of 5 s, the concrete hinge is completely unloaded with a force rate of 5 kN/s. After that, the eccentricity of the concrete hinge is increased to $22\,\mathrm {mm}$ with the help of a special threaded bolt assembly, where turning of screws results in a controlled lateral displacement of the concrete hinge. The test protocol described above is repeated, followed by another increase of the eccentricity to $24\,\mathrm {mm}$ and another repetition of stepwise loading up to $200\,\mathrm {kN}$ and unloading. Thereafter, testing is continued with hours-long creep experiments, as described next.

### Creep under longer-term eccentric compression (175 kN)

Hours-long eccentric compression tests are used to characterize (i) the structural creep behavior under combined compression and bending as well as (ii) the interaction between creep and bending-induced tensile crack propagation. With these two aims in mind, continued testing of concrete hinges was organized as follows.

A 16 h-long creep test is carried out with eccentricity $e=24\,\mathrm {mm}$ and a normal force $N=175\,\mathrm {kN}$. After installing the respective concrete hinge into the testing machine, fine tuning the position, installing the deformation measurement equipment, preliminary loading up to 0.2 kN, removal of the temporary supports, and taking reference readings of the deformation measurement system, loading is increased with a force rate amounting to 5 kN/s up to 175 kN, and this load level is kept constant for 16 h. During this creep test, the DIC system takes 1 picture every 15 min.

## Structural testing of concrete hinges up to their load carrying capacity

A second set of structural tests deals with the load carrying capacity of concrete hinges subjected to eccentric loads. These tests are carried out in order to characterize the relation between eccentric loading and rotation angle, up to structural failure.

### Test setup and measurement equipment

Concrete hinges are subjected, 40 days after production, to compressive line loads using a servo-hydraulic testing machine of type Schenk POZ 0367. The load carrying capacity tests are carried out with an eccentricity of $e =a/3= 25\,\mathrm {mm}$, which refers to the limit of application of the design guidelines of Leonhardt and Reimann [5].

Eight LVDTs of type HBM WI 10 are mounted to the lateral surfaces of the concrete hinges, in order to measure changes of the notch mouth opening displacements of the lateral notches. The measurement length in loading direction amounts to $\ell _\varepsilon ^\mathrm{LCC} =50\,\mathrm {mm}$. The LVDTs are arranged symmetrically in thickness direction with a sensor-to-sensor distance amounting to 100 mm. The measurement axis of each LVDT exhibits a normal distance of 35 mm to the lateral surface of the concrete hinge, i.e. opposite LVDTs exhibit a mutual distance which amounts to $\ell _{\varphi }^\mathrm{LCC} = 250\,\mathrm {mm}+2\cdot 35\,\mathrm {mm}= 320\,\mathrm {mm}$.

### Load carrying capacity tests

In eccentric compression tests ($e = 25\,\mathrm {mm}$) the compressive normal force *N* is increased up to the load carrying capacity. After installing the respective concrete hinge into the testing machine, fine tuning the position, installing the deformation measurement equipment, preliminary loading up to 1 kN, removal of the temporary supports, and taking reference readings of the deformation measurement system, the line-load is increased. This is carried out by manually increasing the displacement of the load application system, with a target rate amounting to 0.2 mm/min. Once 100 kN is reached, the loading process is stopped for 1 min, in order to quantify the time-dependent structural response at that load level. After that, loading is increased by another 100 kN, followed by the next waiting period. Step by step loading is increased up to the load carrying capacity. The latter is assumed to be reached once the rotation angle increases significantly while no significant increase of the normal force is observed. Unloading is performed by using again the rate of 0.2 mm/min. The duration of one test is approximately 30 min.

## Test evaluation and results

### Postprocessing of LVDT measurements

Individual LVDT readings are used to quantify the shortening and the rotation angle of the neck region. To this end, readings on both sides (left and right) are averaged

13 $$\begin{aligned} \Delta l_\mathrm{left}&=  {} \frac{1}{n_s} \sum \limits _{i=1}^{n_s} \Delta l_{\mathrm{left},i}(t) \end{aligned}$$

14 $$\begin{aligned} \Delta l_\mathrm{right}&=  {} \frac{1}{n_s} \sum \limits _{i=1}^{n_s} \Delta l_{\mathrm{right},i}(t) \end{aligned}$$

where $n_s$ denotes the number of sensors mounted on each side of the concrete hinges.^1^ The shortening of the neck region, $\Delta l$, is quantified as

15 $$\begin{aligned} \Delta l = \frac{\Delta l_\mathrm{left}+\Delta l_\mathrm{right}}{2} \end{aligned}$$

and the rotation angle as

16 $$\begin{aligned} \Delta \varphi = \left| \frac{\Delta l_\mathrm{left}-\Delta l_\mathrm{right}}{\ell _{\varphi }}\right| \end{aligned}$$

where $\ell _{\varphi }$ denotes the distance of opposite LVDTs, see Sects. 4.1 and 5.1.

### Material creep tests

**Fig. 5:**
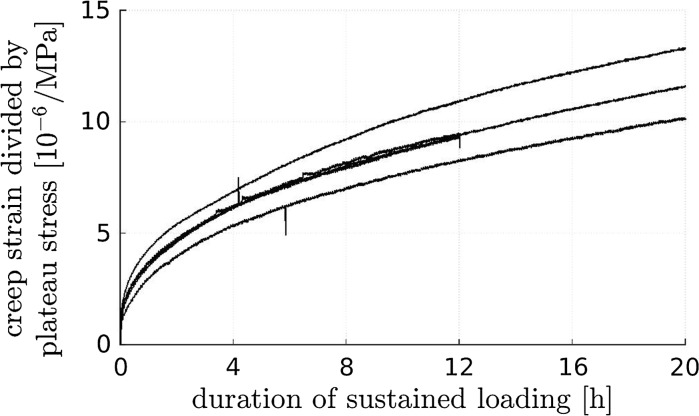
Evolution of creep strains of concrete under constant load ($0.2\cdot f_c$): results from testing of five plain concrete specimens

The five creep tests performed under $\approx 20$ % of the compressive strength yield creep strain evolutions reminiscent of a power-law, see Fig. 5. Three out of five tests delivered virtually the same creep response, framed by the results of the other two tests. This indicates a satisfactory reproducibility of the experiments.

### Centric compression of concrete hinges (200 kN)

**Fig. 6:**
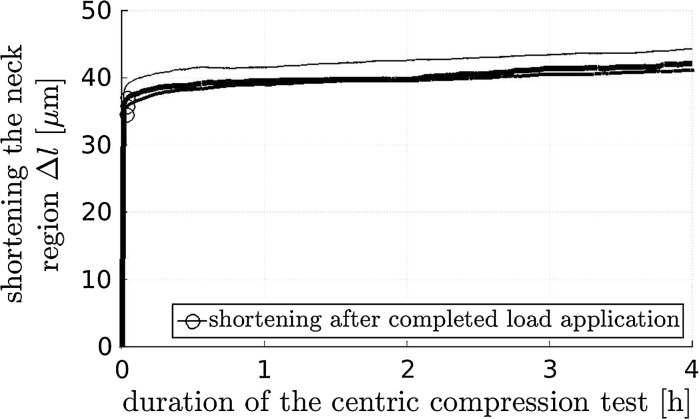
Measured shortenings of the neck regions of three concrete hinges as a function of time; loading is increased with a force rate of 5 kN/s up to 200 kN and kept constant thereafter, demonstrating structural creep under centric compression

All three concrete hinges showed virtually the same behavior during the creep tests. The force-shortening relationships during 40 s-long loading are practically linear. The shortening of the neck, just after arriving at 200 kN, amounts to $\approx 35\,\upmu$m, see the circles in Fig. 6. During the subsequent 4 h-long creep period, the shortening increases nonlinearly by some 20 %, again reminiscent of a power-law creep evolution, see Fig. 6.

### Bending stiffness of concrete hinges and crack propagation during short-term eccentric compression tests up to 200 kN

**Fig. 7:**
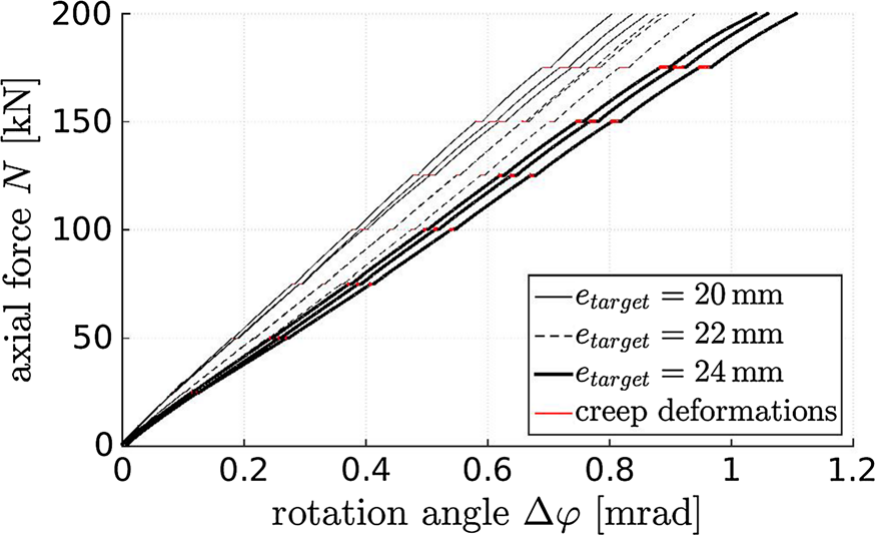
Measured rotation angles of three concrete hinges as a function of eccentricity and loading; loading is increased with a force rate of 5 kN/s and held constant every 25 kN for 10 s; creep deformation developing during these intermediate load plateaus are highlighted in red color. (Color figure online)

Increasing eccentric loading in steps of 25 kN and interrupting the loading process for 5 s at each completed loading step, demonstrates that concrete hinges are very creep active even under small degrees of utilization (relative to the load carrying capacity), see the visible load plateaus highlighted in red color in Fig. 7. Subtracting rotation angles developing during the 5 s waiting periods from the total measurements, would result in rotation angles that increase virtually linearly with increasing loading, with a slope that decreases with increasing eccentricity.

**Fig. 8:**
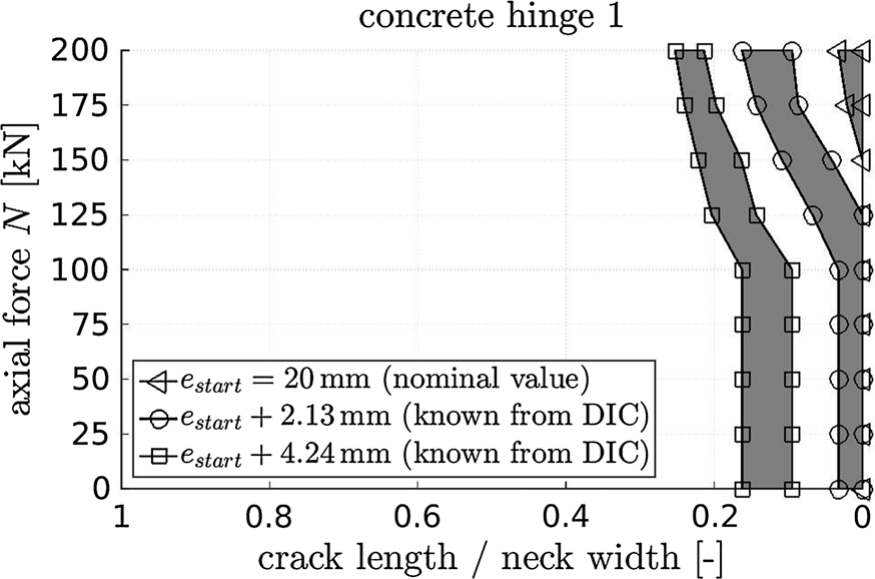
Measured average crack length (=mean of front-side and back-side crack length) of the concrete hinge 1 determined from DIC with settings according to Table 2, with nominal eccentricities of 20, 22, and 24 mm, as well as loading up to 200 kN

When it comes to identification of crack length at the different load plateaus, we consider a region to be cracked, if the maximum strain in loading direction is larger than a threshold. Definition of the latter is a challenging task given the small strain amplitudes before tensile cracking. We here defined an upper and a lower threshold which are expected to be bounds containing the real crack initiation strain, see Table 2. Already with the first eccentricity, nominally amounting to 20 mm, first cracks developed at load levels amounting to 175 and 200 kN, see the triangles in Fig. 8.^2^ After unloading, the eccentricity was increased by nominally 2 mm. Since DIC allows for quantifying rigid body motions, the actual lateral movement could be shown to be slightly larger, i.e. 2.13 mm for concrete hinge 1. When reloading with the increased eccentricity to the first intermediate load level of 25 kN, the already existing crack opened visibly. Crack propagation was observed for loading beyond 100 kN, see the circles in Fig. 8. Similar observations were made also with the third eccentricity and the final crack length amounted to some 20% of the neck width, see the squares in Fig. 8.

### Creep and tensile cracking of concrete hinges during longer-term eccentric compression (175 kN)

**Fig. 9:**
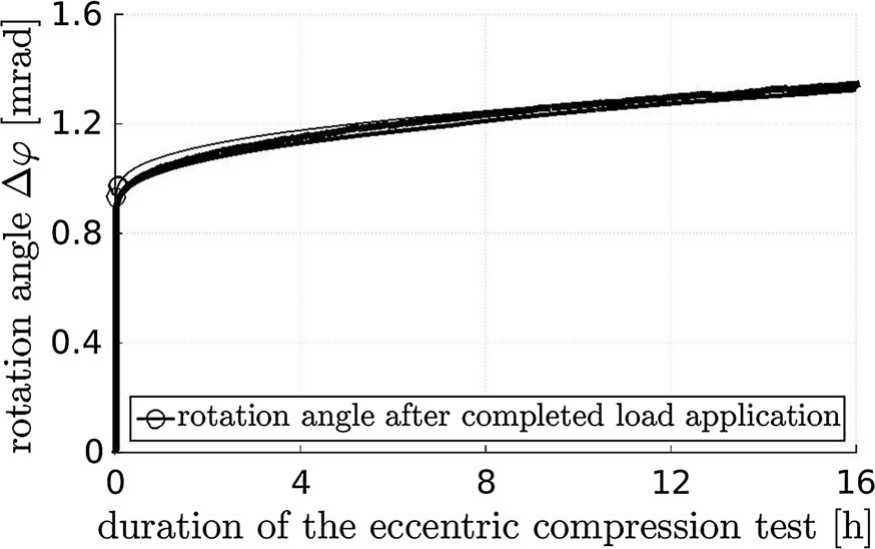
Measured rotation angles of the neck regions of three concrete hinges as a function of time; loading is increased with a force rate of 5 kN/s up to 175 kN and kept constant thereafter, demonstrating structural creep under eccentric load ($e=24$ mm, $N=175$ kN)

All three concrete hinges showed virtually the same behavior during the eccentric creep tests. The relationships between the eccentric force and the rotation angle are practically linear during the loading period amounting to 35 s. The rotation angle of the neck, just after arriving at 175 kN, amounts to $\approx \,0.9$ mrad, see the circles in Fig. 9. During the subsequent 16 h-long sustained eccentric loading, the rotation angle increases nonlinearly by around 50%, again reminiscent of a power-law creep evolution, see Fig. 9.

**Fig. 10:**
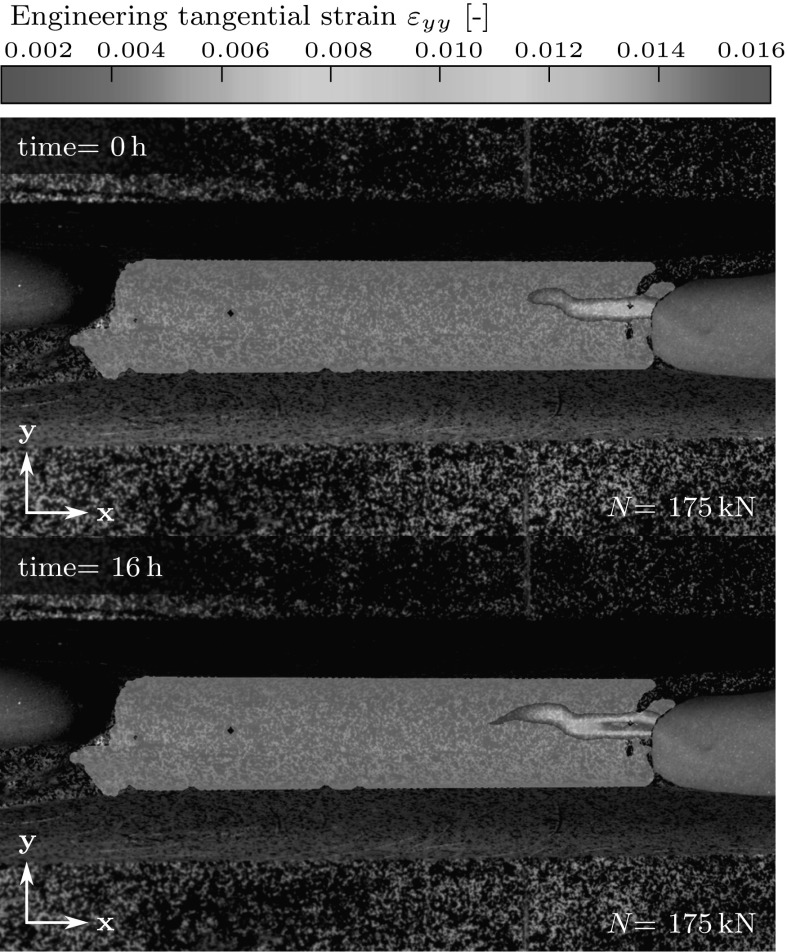
Crack behavior during structural creep at the *back* side notch of concrete hinge 1; eccentricity $e=24$ mm, load $N=175$ kN: strain field in load direction; right after completed load application (time= 0 h) and 16 h later (time= 16 h)

The behavior of the pre-existing crack under constant eccentric loading ($e=24$ mm) is quantified based on DIC images. Just after loading up to 175 kN, the visible cracks are identical to the final cracks observed during short-term eccentric compression tests up to 200 kN. Comparing these initial crack images with DIC images taken 16 h later, it is concluded that the crack mouth opening increases by 52 % (concrete hinge 1) and by 77 % (concrete hinge 3), and that the crack length increases by 16 % (concrete hinge 1) and by 5 % (concrete hinge 3), see Fig. 10. It is noteworthy that the finally obtained crack lengths are by 35 % (concrete hinge 1) and by 32 % (concrete hinge 3) larger than the crack lengths obtained during short-term loading to 175 kN (Fig. 8), i.e. up to the load level of the long-term creep experiment.

### Load carrying capacity of concrete hinges

**Fig. 11:**
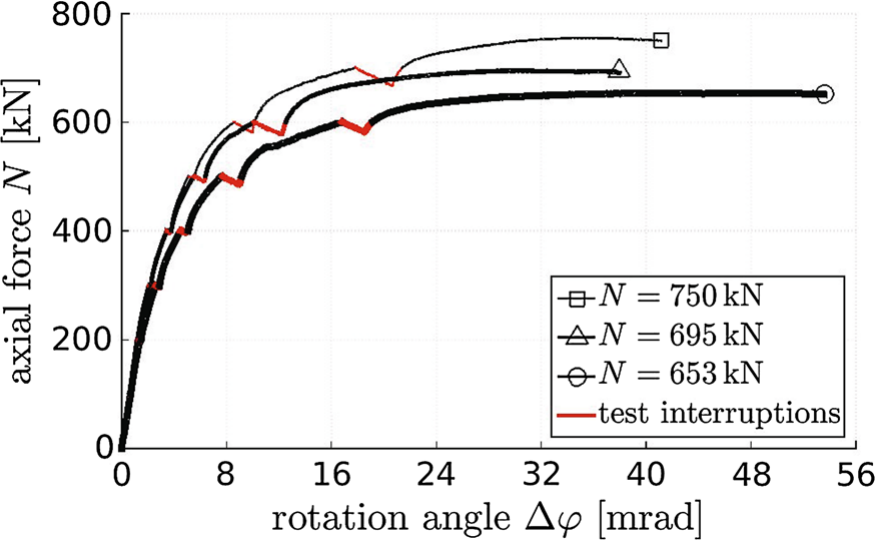
Measured rotation angles of the neck regions of three concrete hinges as a function of eccentric loading ($e=25$ mm) up to the load carrying capacity; viscoelastic deformation developing during short-term test interruptions are highlighted in red color. (Color figure online)

The reached load carrying capacities of the three tested concrete hinges range from 653 to 750 kN, see Fig. 11. The concrete hinges failed in a pronounced ductile fashion, i.e. rotation angles increased for more than 20 mrad while the external loading was practically constant. When interrupting the loading process all 100 kN for 1 min by freezing the piston displacement, the viscoelastic behavior of concrete resulted in increasing rotation and decreasing normal forces, see the red parts of the graphs in Fig. 11. Removing these readings with the aim to obtain graphs which are at least to a certain extent representative for continuous load increase up to the load carrying capacity, would result in quite smooth force-rotation diagrams, indicating a satisfactory reproducibility of the tests.

## Discussion

The following discussion refers to curing and testing conditions (Sect. 7.1), to lessons learned from the numerical re-analysis of the bearing capacity tests (Sect. 7.2), to the interaction between creep and bending-induced tensile cracking of initially monolithic concrete hinges (Sect. 7.3), to implications concerning design guidelines (Sect. 7.4), and to the time-dependent behavior of concrete hinges in integral bridge construction (Sect. 7.5).

### Curing and test conditions

In order to simulate a practical real-life application, all tested specimens were air-cured after formwork removal. Drying resulted in moisture gradients and potentially even in a premature stopping of the hydration process in close-to-surface subvolumes of the specimens. In addition, drying shrinkage strains and autogeneous shrinkage strains of the concrete hinges were restrained by the rebars and the steel plates. Therefore, self-equilibrated stresses (tension in concrete and compression in the steel rebars) developed inside the concrete hinges even before mechanical loading. The tensile stresses may have well resulted in damage of concrete, in form of microcracking. This effect was already pointed out in 1965 by Leonhardt and Reimann [5] for concrete hinges with reinforced necks. A quantitative assessment of pre-existing damage of the tested concrete hinges is provided by the elasto-brittle numerical re-analysis of the bearing capacity tests described in [47], see Sect. 7.2.

### Lessons learned from the numerical re-analysis of the bearing capacity tests

In [47], elasto-brittle numerical simulations (carried out with a commercial Finite Element software for reinforced concrete structures) were combined with a multiscale model for elasticity and tensile strength of concrete. The latter model uses Budiansky and O’Connel’s crack density parameter as the damage variable. Setting it equal to 0.065 allowed for simulating the measured behavior (Figs. 7, 11) very reliably. The corresponding values of the damaged Young’s modulus of concrete, $E_\mathrm{dam}$, and the damaged tensile strength of the material, $f_{t,{\mathrm{dam}}}$, amount to [47]

17 $$\begin{aligned} E_{\mathrm{dam}} = 26.07\,{\text {GPa}}, \quad f_{t,{\mathrm{dam}}} = -3.4\,{\text {MPa}}. \end{aligned}$$

Notably, the two mechanical properties listed in Eq. (17) were found in [47] by fitting. This provides the motivation to check their plausibility based on research results presented herein. Checking is started with the tensile strength value $f_{t,{\mathrm{dam}}}$.

During the short term loading experiments carried out with eccentricity $e=20\,\mathrm {mm}$, cracking of the concrete hinges initiated at an axial force $N=175\,\mathrm {kN}$, see Fig. 8. Inserting these values together with $M=N\cdot e$ as well as with $a=75\,\mathrm {mm}$ and $b = 300\,\mathrm {mm}$ into Eq. (9) delivers an experimentally-derived estimate of the actual tensile strength amounting to $-3.3\,{\text {MPa}}$. This is only slightly different compared to the value found in [47] and, hence, corroborates the value of $f_{t,{\mathrm{dam}}}$ given in Eq. (17). The plausibility of the value of the damaged Young’s modulus of concrete, $E_\mathrm{dam}$, will be checked in the following Sect. 7.3.

### Interaction between creep and bending-induced tensile cracking of concrete hinges

**Fig. 12:**
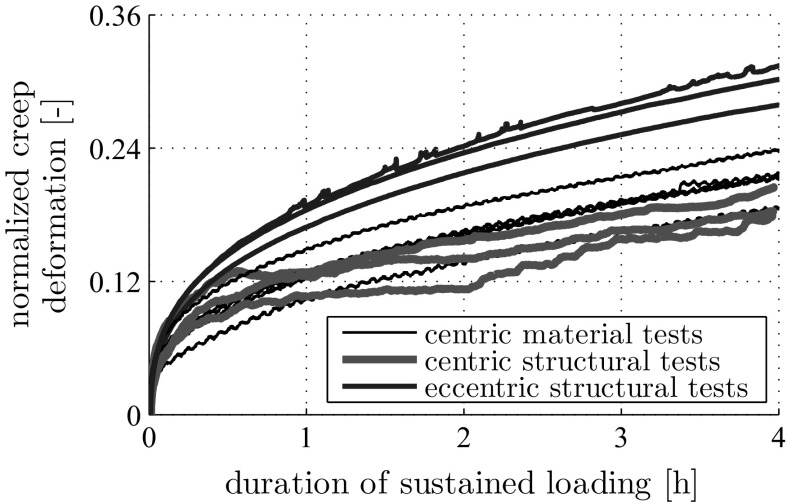
Evolution of creep deformations under sustained loading, normalized by the averaged deformation reached right after completing the loading process

The herein measured material and structural creep properties are qualitatively similar, see the “power-law”-type normalized creep evolutions in Fig. 12. Therein, the ordinates refer to the evolutions of the deformation quantities measured under sustained loading (shortening in centric compression tests, see Figs. 5 and 6, and relative rotation angles in eccentric compression tests, see Fig. 9) divided by the corresponding average deformation quantities measured right after the end of the loading phase.

The material tests and the centric structural experiments delivered also quantitatively very similar normalized creep evolutions, particularly so during the first hour of the tests, see Fig. 12. After this initial phase, load redistributions inside the concrete hinges, i.e. the load transfer from creeping concrete to the non-creeping steel rebars may explain why structural centric creep is by some 10 percent smaller than material creep under centric compression, see Fig. 12.

The described quantitative differences allow for the announced checking of the plausibility of the damaged elastic stiffness value of concrete reported in Eq. (17). In this context, we follow old French regulations (see [48] for a similar approach) and consider two specimens: one made of plain concrete and the second made of reinforced concrete, whereby concrete and steel rebars are arranged in parallel. Also, we envision that uniaxial loading is applied in the rebar direction and that concrete and steel are firmly bonded to each other in the reinforced specimen, such that both materials exhibit the same axial normal strain. Provided that the same force density is applied to both specimens, the stress of plain concrete will be by a factor of $1 + \varrho \,E_s/E_\mathrm{dam}$ larger than the stress of concrete in the reinforced specimen [48], where $E_\mathrm{dam}$ denotes the Young’s modulus of the concrete of the reinforced specimen. Therefore, also the creep activity of the plain concrete specimen can be envisioned to be by the same factor larger than the one of the reinforced specimen. Inserting the values of $\varrho$, $E_s$, and $E_\mathrm{dam}$ according to Eqs. (6), (3), and (17) into the expression $1 + \varrho \,E_s/E_\mathrm{dam}$ delivers a numerical value of the multiplication factor amounting to 1.10. This is consistent with the experimentally observed difference in the creep strains between (i) plain concrete and (ii) reinforced concrete hinges subjected to centric compression, see Fig. 12. Therefore, the value of $E_\mathrm{dam}$ given in Eq. (17) is corroborated.

The by far largest normalized creep activity was measured in the eccentric tests on initially monolithic concrete hinges. This underlines a considerable interaction between creep *and* bending-induced tensile cracking:

- Structural creep under eccentric compression increases both the crack opening displacements and the crack length, see Sect. 6.5.
- Vice versa, progressive crack opening and crack propagation amplify the structural creep activity compared to the one observed in centric creep tests, see Fig. 12.

This result of the present study is consistent with the frequently described expectation that during *relaxation* of concrete hinges under an imposed relative rotation angle (i) cracks will close in the vicinity of the crack tip and (ii) crack opening displacements will decrease elsewhere.

The described creep tests had a characteristic duration ranging from 12 to 20 h. This is negligibly short as compared to 24 days which is the period of drying ranging from stripping of the formworks to the start of the creep tests. Therefore, no significant *additional drying* happened *during* the creep tests and the measured creep behavior refers to basic creep of *pre-dried* specimens. Also, the performed creep tests were very short compared to real application cases. This is the motivation to discuss decades long creep processes in Sect. 7.4. Before that, it is noteworthy for future modeling activities that measured creep activities could be fitted by standard models such as the ones, e.g., suggested by the FIB Model Code [43] or the B4 model [49].

### Consideration of the time-dependent behavior of concrete hinges in available design guidelines

**Fig. 13:**
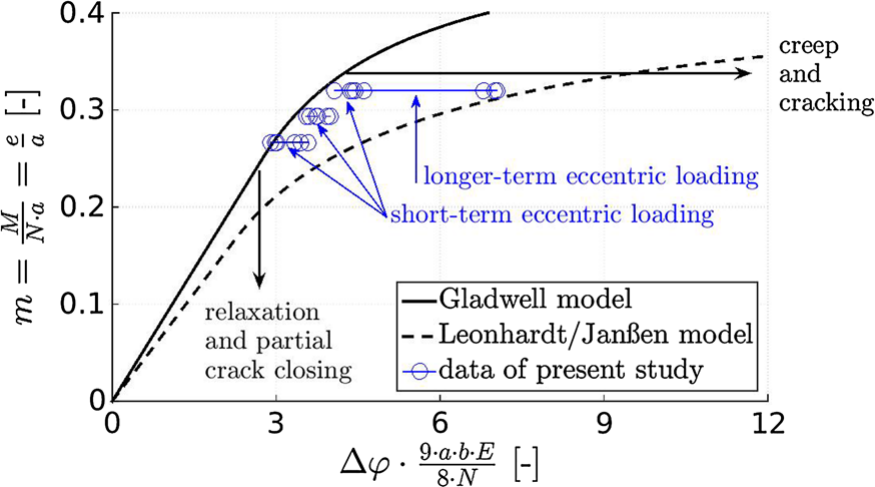
Dimensionless relation between bending moment and relative rotation angle (“Leonhardt and Reimann”-diagram): modeled relationships by Gladwell [30], Leonhardt and Reimann [5], and Janßen [17], as well as experimental data from the present study; see the caption of Fig. 1 for the explanation of the used symbols

Existing design guidelines either disregard the time-dependent behavior of concrete hinges or consider it in a very simplified manner. The model by Gladwell [30] was derived from the elastic contact problem of a flat punch pressed unsymmetrically into a half space. This elasto-brittle model envisions a vanishing tensile strength of concrete and does not consider any time-dependent deformation mechanism. Therefore, it is expected to represent an upper bound for all available experimental data, see Figs. 1 and 13. Leonhardt and Reimann [5], in turn, combined equilibrium conditions with a linear compressive stress distribution in the neck region, i.e. also this approach is based on a vanishing tensile strength. In addition, the model considers time-dependent behavior of concrete in a very simplified manner, i.e. the elastic compliance is increased by a multiplicative factor of 2. In the 1960s, namely, the time-dependent behavior of concrete was considered to come to an end, such that a concrete structure would reach asymptotically a stationary state under permanent loading. Leonhardt and Reimann assumed that the finally reached *total* compliance of concrete, related to both instantaneous elastic and delayed time-dependent deformation, would be twice as large as the instantaneous compliance.

Creep of concrete, however, does not come to an end: it starts with power-law-type creep rates and decays later to logarithmic creep rates which do not approach a stable asymptotic value [46]. This is evidenced by decades long testing of concrete samples [50] and monitoring of the deflections of concrete bridges [51]. It is the motivation to consider bending-related creep and relaxation explicitly in Fig. 13, whereby the normal force is considered to be constant:

- Creep under a sustained bending moment results in a progressive increase of the relative rotation angle, see the abscissa-parallel chains of data points in Fig. 13, referring to short-term and longer-term testing under loads $\le 200$ kN. Because creep of concrete does not come to an end (see above), relative rotation angles must be expected to increase monotonously in a long-term creep test, see the abscissa-parallel arrow in Fig. 13.
- Relaxation under an imposed relative rotation angle, in turn, results in a progressive reduction of the bending moment, see the ordinate-parallel arrow in Fig. 13. Since unbounded creep functions imply that relaxation functions of concrete decay to zero, one may expect that bending moments will finally vanish in a long-term relaxation test.

In integral bridge construction, however, concrete hinges experience bending loads which do not refer to perfect creep or relaxation scenarios, as discussed next for the important exemplary case of frame-type integral bridges.

### Time-dependent behavior of concrete hinges in frame-type integral bridge construction

Once a frame-type bridge is completed, dead load of the structure is transmitted across concrete hinges positioned, e.g., at the feet of the columns. Therefore, each concrete hinge is subjected to a compressive normal force and to a bending moment. The viscoelastic behavior of concrete results in a progressively increasing rotation angle at the neck of the concrete hinge. As a feedback from the statically indeterminate structure, the bending moment at the concrete hinge decreases. In other words, the time-dependent behavior of concrete hinges results in load redistributions within the bridge structure, such that—asymptotically—a vanishing bending moment at the concrete hinge will be obtained. Therefore, modeling concrete hinges as classical hinges without bending stiffness is a well-suited approach for the simulation of the finally reached structural state under permanent loading. The corresponding normal forces, however, have to be transmitted across the concrete hinges permanently. In order to avoid the risk of nonlinear creep in compression, i.e. the interaction between creep and progressive damage [52–54], it is recommended—in agreement with Eurocode 2 regulating the design of concrete bridges, see [44]—(i) to limit the permanent compressive normal forces with 45 % of the corresponding ultimate load carrying capacity of the concrete hinge, and (ii) to treat load redistributions from creeping concrete to non-creeping steel rebars as hidden reserves.

Variable loads, such as the ones resulting from daily changes of the ambient temperature, exhibit characteristic times which are equal to the ones studied in the present creep experiments. This implies that modeling concrete hinges as rotational springs with a time-dependent stiffness is required for realistic structural simulations in integral bridge construction. The herein characterized mechanical properties of concrete and the experimental data obtained in the structural tests are well suited to support these future modeling activities. In this context, it is noteworthy that the structural tests with forces $\le 200$ kN are representative for both reinforced and unreinforced concrete hinges, because of the small reinforcement ratio, see Eq. (6), and because the reinforcements were compressed, as is evidenced from crack lengths which are significantly smaller than half of the neck width.

## Conclusions

From the obtained experimental data and from the related discussions, the following conclusions are drawn:

- Concrete hinges are very creep active structures. The creep-related shortening of the neck under centric compression increased in 4 h by 20 % relative to the shortening developing during loading (Fig. 6). The creep-related rotation angle of the neck under eccentric compression increased in 16 h by 50 % relative to the rotation angle developing during loading (Fig. 9).
- Creep and bending-induced tensile cracking of concrete hinges are interacting phenomena. Creep under eccentric compression increases the length and opening displacements of bending-induced tensile cracks. The increased crack length, in turn, amplifies the structural creep activity relative to material or structural creep under centric compression (Fig. 12).
- Vice versa, stress relaxation will result in partial crack closure and in decreasing crack opening displacements, which is very beneficial for the durability of concrete hinges.

As for the simulation of initially monolithic concrete hinges in frame-like integral bridge construction, the following conclusions are drawn:

- As for permanent loads, modeling of concrete hinges as classical hinges *without* bending stiffness is a sound approach for assessing the finally reached structural state.
- In order to avoid progressive damage under permanent compressive normal forces, the latter shall be limited to 45 % of the corresponding ultimate load carrying capacity of the concrete hinge.
- As for variable loads, future modeling of concrete hinges as rotational springs with *time-dependent* stiffness is required for reliable simulations.
- The presented experimental data may well support such future modeling activities.

## Footnotes

1 One of the outermost LVDTs failed during one test with $N \le 200$ kN. In the interest of comparability of different tests, $n_s$ is set equal to 3 referring to the three central LVDTs of each side.

2 DIC images of concrete hinge 2 could not be evaluated due to insufficient contrast. Results regarding concrete hinge 3 are similar to the ones of concrete hinge 1, but they cannot be shown due to the limited number of allowed figures.
